# Genomic alterations identified by array comparative genomic hybridization as prognostic markers in tamoxifen-treated estrogen receptor-positive breast cancer

**DOI:** 10.1186/1471-2407-6-92

**Published:** 2006-04-12

**Authors:** Wonshik Han, Mi-Ryung Han, Jason Jongho Kang, Ji-Yeon Bae, Ji Hyun Lee, Young Ju Bae, Jeong Eon Lee, Hyuk-Jae Shin, Ki-Tae Hwang, Sung-Eun Hwang, Sung-Won Kim, Dong-Young Noh

**Affiliations:** 1 Department of Surgery, Seoul National University College of Medicine, Seoul, Korea; 2 Cancer Research Institute, Seoul National University College of Medicine, Seoul, Korea; 3 Macrogen Inc., Seoul, Korea

## Abstract

**Background:**

A considerable proportion of estrogen receptor (ER)-positive breast cancer recurs despite tamoxifen treatment, which is a serious problem commonly encountered in clinical practice. We tried to find novel prognostic markers in this subtype of breast cancer.

**Methods:**

We performed array comparative genomic hybridization (CGH) with 1,440 human bacterial artificial chromosome (BAC) clones to assess copy number changes in 28 fresh-frozen ER-positive breast cancer tissues. All of the patients included had received at least 1 year of tamoxifen treatment. Nine patients had distant recurrence within 5 years (Recurrence group) of diagnosis and 19 patients were alive without disease at least 5 years after diagnosis (Non-recurrence group).

**Results:**

Potential prognostic variables were comparable between the two groups. In an unsupervised clustering analysis, samples from each group were well separated. The most common regions of gain in all samples were 1q32.1, 17q23.3, 8q24.11, 17q12-q21.1, and 8p11.21, and the most common regions of loss were 6q14.1-q16.3, 11q21-q24.3, and 13q13.2-q14.3, as called by CGH-Explorer software. The average frequency of copy number changes was similar between the two groups. The most significant chromosomal alterations found more often in the Recurrence group using two different statistical methods were loss of 11p15.5-p15.4, 1p36.33, 11q13.1, and 11p11.2 (adjusted *p *values <0.001). In subgroup analysis according to lymph node status, loss of 11p15 and 1p36 were found more often in Recurrence group with borderline significance within the lymph node positive patients (adjusted *p *= 0.052).

**Conclusion:**

Our array CGH analysis with BAC clones could detect various genomic alterations in ER-positive breast cancers, and Recurrence group samples showed a significantly different pattern of DNA copy number changes than did Non-recurrence group samples.

## Background

The incidence of breast cancer has been rapidly increasing in Korea and it has been the most frequent malignancy in Korean women since 2002 [1]. Breast cancer is a highly heterogeneous disease both histologically and molecularly, and hormone receptor-positive and -negative tumors are quite distinct biologically. Recent gene expression profiling has identified hormone receptors as a fundamental parameter for distinguishing breast cancers, suggesting a molecular difference according to hormone receptor status [2]. More than 50% of breast cancer cases are estrogen-dependent, and treatment with estrogen antagonists that inhibit estrogen receptor (ER) action, particularly tamoxifen, has contributed to a dramatic reduction in breast cancer mortality. However, a substantial proportion of patients expressing ER either fail to respond initially or become progressively resistant to endocrine therapies [3]. Thus, it would be ideal to predict therapeutic efficacy for each patient before treatment is initiated.

Genetic and epigenetic alterations are important steps in the development of malignancies and may contribute to disease progression during treatment. Similarly, genetic alterations may play a role in the development of tamoxifen resistance [4]. Searches for genes exhibiting altered expression in resistant breast cancer cells have been performed using differential display, Serial Analysis of Gene Expression (SAGE), Comparative Genomic Hybridization (CGH), and expression microarray, and several marker genes have been identified using these techniques [5-9]. However, the exact molecular mechanism, other than ER expression, underlying tamoxifen response and resistance is not yet understood.

Array CGH has been used to localize copy number changes associated with human breast and other cancers [10-14]. Similar to chromosomal CGH, array CGH compares the abundance of specific genomic sequences in whole-tumor DNA relative to normal reference genomes. Array CGH can provide higher resolution than conventional CGH with more accurate mapping of regions that contain oncogenes or tumor suppressor genes [15]. With more and more array CGH data emerging, there is a need for efficient algorithms that automatically select regions of gains and losses. Recently, various software items have been released to make this complex analysis possible [16-21].

In this study, we used array CGH to assess DNA copy number changes in 28 fresh-frozen ER-positive breast cancer tissue samples. A program for array CGH data analysis, the CGH-Explorer and Analysis of Copy Error (ACE) algorithm by Lingjærde *et al*. [20], was used for calling gains and losses. The purpose of this study was to elucidate whether DNA copy number changes in the primary tumor can predict a patient's prognosis and tamoxifen responsiveness in ER-positive breast cancer and to identify the corresponding chromosomal regions and genes.

## Methods

### Patients and tumor specimens

A total of 28 primary invasive breast cancer tissues selected from the frozen tissue archives in the Cancer Research Institute, Seoul National University, were used in this study. All tumors were excised between November 1996 and February 2001 and were histopathologically confirmed as invasive ductal carcinoma. No *in situ *cancers were included. This study was conducted under the approval of the Institutional Review Board of Seoul National University Hospital. Informed consent was obtained from all participants prior to surgery. All patients received tamoxifen as an adjuvant endocrine therapy for at least 1 year. No other type of hormone therapy was used during the follow-up period. Nine patients had distant metastasis within 5 years of diagnosis (Recurrence group), and 19 patients were alive without evidence of disease at least 5 years after diagnosis (Non-recurrence group). Patients in the Recurrence group experienced distant metastasis at 16 to 53 months after diagnosis (mean, 33 months). Surgical procedures did not differ substantially between the two groups: modified radical mastectomy was performed in 7 patients (77.8%) in the Recurrence group and 17 patients (89.5%) in the Non-recurrence group. The remaining patients in each group underwent breast-conserving surgery plus adjuvant radiotherapy. Most patients received adjuvant chemotherapy after surgery, except for one patient in the Non-recurrence group. Chemotherapy regimens included six cycles of CMF (cyclophosphamide, methotrexate, and 5-fluorouracil) in 17 patients (63%) and a doxorubicin based regimen ± a taxane in 10 patients (37%). Median follow-up time for survival analysis was 66 months. Patient age at diagnosis, histologic subtype, tumor size, lymph node status, histological grade (Scarff-Bloom-Richardson classification), and nuclear grade (Black's nuclear grade) were reviewed. Immunohistochemical staining (IHC) was performed to determine expression of the ER and progesterone receptor (PR). The primary antibodies used for ER and PR (DAKO, Glostrup, Denmark) and immunohistochemical methods have been previously described [22]. A cut-off value of ≥ 10% positively-stained cells per 10 high-power fields was used to determine ER and PR positivity. Tissue samples were frozen in liquid nitrogen within 20 minutes following devascularization in the operating room and stored at -80°C. All tumor specimens analyzed contained more than 50% tumor cells.

### Construction of BAC library

The array used in this study consists of 1,440 human Bacterial Artificial Chromosomes (BACs), which were spaced approximately 2.3 Mb on average across the entire genome (MacArray™ Karyo1400 from Macrogen, Inc., Seoul, Korea). BAC clones were selected from the proprietary BAC library of Macrogen, Inc. Briefly, the pECBAC1 vector [23] was digested with HindIII and size-selected HindIII-digested pooled male DNAs were used to generate a BAC library. These vectors were then transformed into and grown in the *Escherichia coli *DH10B strain.

### Construction of BAC-mediated array CGH microarray

Clones were first selected to yield an average genomic coverage of 2-Mb resolution. All clones were two-end sequenced using an ABI PRISM^® ^3700 DNA Analyzer (Applied Biosystems, Foster City, CA, USA), and their sequences were blasted and mapped according to their positions described in the University of California, Santa Cruz (UCSC) human genome database . Confirmation of locus specificity of the chosen clones was performed by removing multiple loci-binding clones by individual examination under standard fluorescence in situ hybridization (FISH) as described previously [24]. These clones were prepared by the conventional alkaline lysis method to obtain BAC DNA. The DNA was sonicated to generate ~3-kb fragments before mixing with 50% DMSO spotting buffer. The arrays were manufactured by an OmniGrid arrayer (GeneMachine, San Carlos, CA, USA) using a 24-pin format. Each BAC clone was represented on an array as triplicate spots and each array was pre-scanned using a GenePix 4200A scanner (Axon Instruments Inc., Foster City, CA, USA) for proper spot morphology.

### DNA labeling for array CGH

DNA was extracted from each breast cancer tissue sample using the PureGene kit (Gentra Systems Inc., Minneapolis, MN, USA). The labeling and hybridization protocols described by Pinkel *et al*. [15] were used with some modification to the labeling procedure. Briefly, 21 μl solution containing 500 ng normal DNA (reference DNA) or tumor DNA (test DNA), 20 μl BioPrime^® ^DNA Labeling System random primers solution (Invitrogen, Carlsbad, CA, USA), and water were combined and incubated for 5 min at 95°C, and subsequently cooled on ice. After the addition of 5 μl 10 × dNTPs labeling mix (1 mM dCTP, 2 mM dATP, 2 mM dGTP, 2 mM dTTP), 3 μl 1 mM Cy-3 or Cy-5 dCTP (GeneChem Inc., Daejeon, Korea), and 40 U BioPrime^® ^DNA Labeling System Klenow fragment (Invitrogen), the mixture was gently mixed and incubated overnight at 37°C. The addition of 5 μl BioPrime^® ^DNA Labeling System Stop Buffer (Invitrogen) ended the reaction. After labeling, unincorporated fluorescent nucleotides were removed using QIAquick Spin columns (Qiagen, Germany). In one tube, Cy3-labeled sample and Cy5-labeled reference DNAs were mixed together, and 50 μg human Cot I DNA (Invitrogen), 20 μl 3 M sodium acetate, and 600 μl cold 100% ethanol were added for DNA precipitation.

### Array hybridization, imaging, and data preprocessing

The pellet was resuspended in 40 μl hybridization solution containing 50% formamide, 10% dextran sulfate, 2× SSC, 4% SDS, and 200 μg yeast tRNA. The hybridization solution was denatured for 10 min at 72°C and was subsequently incubated for 1 hour at 37°C to allow blocking of repetitive sequences. Hybridization was performed in slide chambers for 48 hours at 37°C. After post-hybridization washes, arrays were rinsed, spin-dried, and scanned into two 16-bit TIFF image files using a GenePix 4200 A two-color fluorescent scanner (Axon Instruments), and individual spots were analyzed with GenePix Pro 3.0 imaging software (Axon Instruments). Clones on the X and Y chromosomes were not used for further analysis because their intensity may distort the entire data set. The log_2_-transformed fluorescent ratios were calculated from background-subtracted median intensity values, and these ratios were used to perform normalization according to intensity normalization methods. We applied LOWESS normalization, a smoothing adjustment that removes intensity-dependent variation in dye bias [25]. Data for each slide was normalized so that the mean in-slide expression value was 0 and the SD was 1. 109 different BAC clones have been excluded from further analysis because values of at least 2 spots of the triplicate have been missing. Only triplicate of 1,331 different BAC clones were used.

### Real-time quantitative polymerase chain reaction (PCR)-based copy number validation

The DNA copy number of one clone, BAC57_O15 (8q21.13; BAC start 82359567 and end 82463826: NCBI homo sapiens genome Build 35.1) was assayed according to a previously described protocol with normalized normal human pooled genomic DNA (Promega, Madison, WI, USA) as a calibration sample. Real-time PCR reactions were performed on an ABI 7900HT (Applied Biosystems). Amplification mixtures (20 μl) contained template DNA (approximately 2 ng), 1 × TaqMan PCR MasterMix, and 900 nM of each primer. PCR cycling conditions comprised 10 min polymerase activation at 95°C, 40 cycles at 95°C for 15 sec, and 60°C for 1 min. The relative copy numbers of the selected chromosomal locations were determined in the tumor samples and on three normal control DNAs using the same primer according to the manufacturer's protocol, and the copy numbers were normalized based on the standard curve generated from those three control samples.

### Statistical analysis

The average ratio of the three replicate spots for each clone was calculated. A total of 1,331 different BAC clones were used in the final analysis. First, we performed complete linkage hierarchical clustering based on Manhattan distance measure of all 28 samples using the normalized 1,331 clones [26]. This processed data set was then subjected to copy number change analyses for identification of regions of amplification and deletion. For calling gains and losses in array CGH data, the Analysis of Copy Errors (ACE) algorithm in CGH-Explorer [20] was used with an False Discovery Rate (FDR) <0.01. CGH-Explorer was also used for graphical illustration of CGH data. Recently, Lai et al. compared 11 different algorithms which are most frequently used for analyzing array CGH data [27]. In this paper, they pointed out that some current implementations does not include any assessment of the statistical significance of the reported copy number changes, although quantitative statistics about the aberrations are critical in order to decide which region to pursue for further analysis. ACE is one of only two algorithms that incorporate FDR so far. Another advantage of ACE is that it can be used even in situations where normal DNA is unavailable.

The counts of loss or gain vs. no change were summarized by tumor group for each BAC, providing 2 x 2 tables for analysis. Chi-square analysis was applied to these tables to test for a significant difference in the distribution of loss or gain vs. no change between tumor groups (Recurrence and Non-recurrence) for each BAC. We then used the *q *value from the R package of Bioconductor to adjust for multiple comparisons and assign these resulting *q *values as adjusted *p *values. Equivalently, the *q *value is the minimal FDR at which the gene/clone appears significant.

As an alternative analysis to increase our power in identifying regional changes in copy number between tumor groups (Recurrence and Non-recurrence), we averaged log_2 _ratios over a window of three consecutive BACs, shifting along the chromosome one BAC at a time. To find clones with differential aberrations between groups, we used the Significance Analysis of Microarrays (SAM) software [28] for the averaged log_2 _ratios. SAM is most effective for small numbers of experiments and is the most popular method employed for microarray analysis. In the microarray context, the expression levels of some genes are highly correlated although an analytical FDR approach assumes that tests are independent. To overcome this drawback, SAM uses permutations to get an estimate for the FDR of the reported differential genes. SAM score (*d*) is the *T*-statistic value. "Fold change" in SAM output is the ratio of average expression levels of a given gene under each of two conditions (Recurrence group/Non-recurrence group in this study). SAM adopts *q*-value as the lowest FDR at which the gene is called significant. The *q*-value measures how significant the gene is: as *d *> 0 increases, the corresponding *q*-value decreases.

The follow-up duration was calculated from the date of diagnosis until the date of death or last contact. The distant metastasis-free survival was the time between diagnosis and confirmation of distant recurrence. Survival estimates were computed using the Kaplan-Meier method and the differences between survival times were assessed by means of the log rank test. Multivariate analyses were carried out using Cox's proportional hazards model [29]. Survival analyses were carried out using the SPSS (version 12.0) software package (Chicago, IL, USA).

## Results

The distribution of potential prognostic factors was roughly matched between the Recurrence and Non-recurrence groups. Patient age, tumor size, axillary lymph node metastasis, histologic grade, nuclear grade, and expression of PR were not significantly different between the two groups, as shown in Table 1.

**Figure 1:**
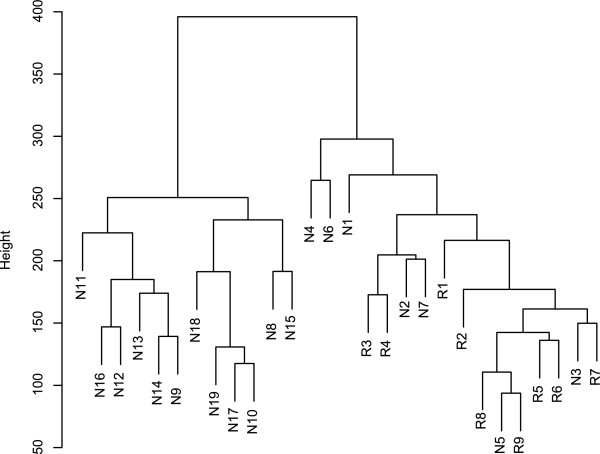
**Cluster dendrogram of array CGH data from 28 ER (+) breast tumor tissues by unsupervised clustering. **The scale on the left vertical bar indicates Manhattan distance. Samples with the prefix R- belong to the Recurrence group and those with the prefix N- belong to the Non-recurrence group. The samples from the nine patients in the Recurrence group were clustered together under the third-order dendrogram branch.

First, we performed complete linkage unsupervised hierarchical clustering of all 28 samples using the 1,331 clones distributed across the 22 autosome pairs. The nine samples from Recurrence group patients tended to be located relatively close together below the third-order branch in the dendrogram (Figure 1). The genomic alteration pattern was expected to differ considerably between the Recurrence and Non-recurrence groups in this analysis.

**Figure 2:**
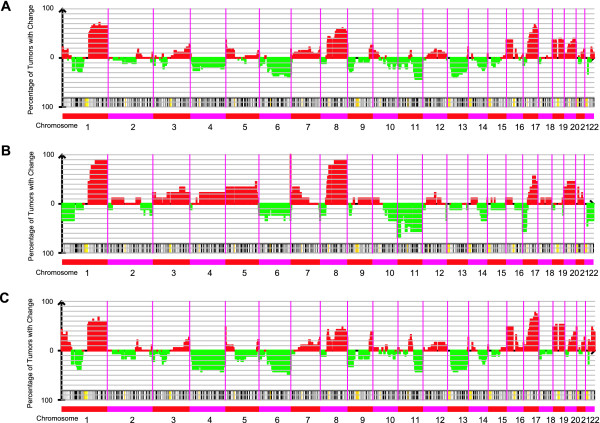
**Frequency plots of gains and losses of each chromosomal region in all samples (A), Recurrence group samples (B), and Non-recurrence group samples (C). **Red/green colored bars correspond to the percentage of samples with gain or loss in the given chromosomal region.

Using the ACE algorithm in the CGH-Explorer program, copy errors were determined as either copy gain or loss. Out of the 1,331 clones, 1,154 clones showed gains and 1,088 clones showed losses in at least one sample. Only 14 clones showed no gain or loss across all 28 samples. The chromosomal regions with common genomic alterations (≥ 20%) are shown in Table 2. Gains in 1q21.1-q44, 17q23.2-q25.3, 8q11.21-q24.3, 17q12-q22, and 8p12-p11.1, and losses in 6q11.1-q27 and 11q13.5-q24.3 were the most common altered regions among the 28 ER-positive breast cancer samples (Table 2 and Fig. 2A). The majority of these regions have been reported in previous conventional or array CGH breast cancer studies [13,14,30,31].

To identify genomic alterations that are associated with disease recurrence, we first compared the gain and loss frequencies between the Recurrence and Non-recurrence groups. The average frequency of copy number changes was similar between the two groups. Of the Recurrence group samples, an average of 14.7% of the clones had gains and 12.3% had losses, whereas an average of 16.0% and 12.8% of the clones in the Non-recurrence group samples had gains and losses, respectively. The frequency patterns of copy number changes for each group across the entire genome are shown in Fig. 2B and 2C.

**Figure 3:**
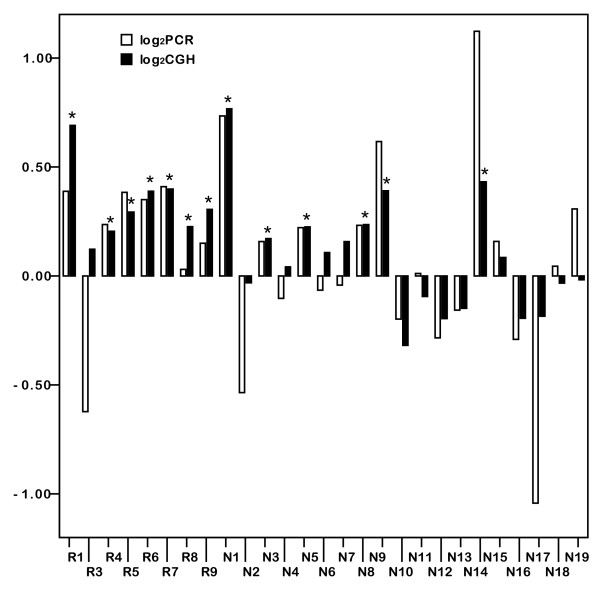
**Correlation of array CGH data (black bar) with real-time PCR data (white bar). **Vertical axis is log_2 _ratio of each sample DNA to control. *, samples called as "gain" with CGH-Explorer. The samples called as gain in array CGH study tend to have higher value with real-time PCR than the other samples.

Using the chi-square test applied to a 2 × 2 table to compare gain and loss of individual BACs between the Recurrence and Non-recurrence groups, we identified BACs that were significantly different between the two subtypes (adjusted *p *value <0.05), including gains in 8q21.13, 19p13.12-p13.11, 19q12-q13.42, 5q11.2-q14.3, 8q24.22, and 8q12.1, and losses in 11p15.5-p15.2, 22q12.3, 1p36.33-p36.11, 11q13.2-q13.5, and 17p13.1 (Table 3 and 4). Using a more stringent threshold of significance (adjusted *p *<0.01), loss in 11p15.5-p15.4 was the only difference between the Recurrence and Non-recurrence groups. Gains in 19p, 19q, 22q, 1p, 9q, and 17q and loss in 4q were found more often in the Non-recurrence group than in the Recurrence group. To validate our array CGH data, real-time PCR for 8q21.13 was performed, which revealed a correlation between the two methods (Figure 3).

As an additional method to identify copy regional changes that differed significantly between the two groups, we used SAM analysis applied to the sliding windows of three consecutive BAC clones from each group. The majority of significant changes were toward loss in the Recurrence group or gain in the Non-recurrence group. Copy changes of 125 clones were different with *q *values in SAM <0.5% between the two groups, including 11p15, 1p36, 7q22, 11q13, 19p13, 19q13, 9q34, 3q21, 10q26, and 11p11 (Table 5). Among these, losses in 11p15.5-p15.4, 1p36.33, 11q13.1, and 11p11.2 were significantly more frequent in the Recurrence group using both types of analysis. These regions that exhibited copy number changes are therefore strong candidates for prognosis indicators in ER-positive breast cancers.

**Figure 4:**
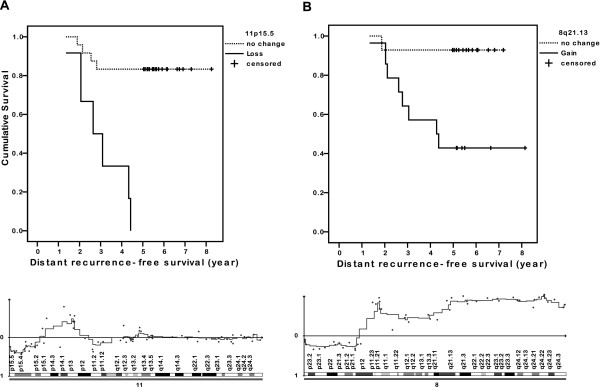
**Top, Distant recurrence-free survival curves for all 28 patients according to whether or not they have 11p15.5 loss (A) and 8q21.13 gain (B). **Bottom, Point plot and moving average (solid line) of representative samples showing 11p15.5 loss and 8q21.13 gain.

In the Kaplan-Meier analysis of the 28 patients, both loss at 11p15.5 and gain at 8q21.13 were significantly associated with distant metastasis-free survival (*p *<0.001 and *p *= 0.006, respectively; Fig 4A, B). 1p36.33 loss was also the significant prognostic factor for distant recurrence (p <0.001, figure not shown). Multivariate analysis using the Cox proportional hazard model with parameters including age, T stage, lymph node status, nuclear grade, and PR revealed that loss of 11p15.5 was the most significant factor among the different variables (hazard ratio 12.3 [95% confidence interval: 2.7–55.4]) (Table 6).

Next, we did subgroup analysis according to lymph node status. The number of cases in lymph node negative patients was too small for this analysis (n = 2 in Recurrence group and N = 6 in Non-recurrence group). In lymph node positive patients, no chromosomal aberration was significantly different between the two risk groups with an adjusted *p *value <0.05. Loss of 1p36.33-p35.3 and 11p15.5-p15.4 were found more often in Recurrence group, and loss of 4q13.1-q34.3 and gain of 19q12-q13.2 were found more in Non-recurrence group with borderline significance (*p *= 0.052) (Table 7).

## Discussion

This study showed that genomic alteration patterns detected by array CGH are different between ER-positive breast cancer patients in the Recurrence group and the Non-recurrence group after surgery and tamoxifen treatment. These differences were first suggested by unsupervised hierarchical clustering and then confirmed by two statistical methods that were designed to identify the most different specific BAC clones and chromosomal regions between the two groups. We found that loss of 11p15 and 1p36 and gain of 8q21 are significantly associated with distant recurrence of the disease within 5 years of diagnosis. These chromosomal aberrations are thus candidate markers for tumor aggressiveness or tamoxifen resistance in ER-positive breast cancers. The independent prognostic significance of two of these chromosomal aberrations was further demonstrated by a striking survival difference in patients who did and did not have the corresponding chromosomal copy number change. The fact that most of the genomic alterations that we identified have been previously reported in other conventional or array CGH studies underscores the confidence of the methodology used here. Clinically, if we can predict the prognosis of ER-positive breast cancer by whole genome DNA copy number analysis, use of new generation aromatase inhibitors or cytotoxic chemotherapy can be actively considered for the patients with a poorer prognosis.

Allelic loss at 11p15.5 is frequently observed in a variety of tumors, including breast cancer [32-35]. In invasive ductal carcinoma, the frequency of LOH at 11p15.5 is approximately 30–60% [32,33]. Two distinct regions on chromosome 11p15 that are subjected to LOH in breast cancer have been identified and refined by Karnik et al. [34]. Phillips et al. determined the potential effects of chromosome 11 on the tumorigenic and metastatic abilities of the MDA-MB-435 cell line via chromosome transfer, and indicated that chromosome 11 harbors a metastasis-suppressor gene for breast cancer [36]. Until now, any association between chromosome 11p15.5 and clinical parameters such as recurrence or survival has not been well established. Several studies have suggested that LOH in this region increases during breast cancer progression [34,37,38], and one conventional CGH study showed that 11p loss is associated with disease recurrence in lymph node-negative breast cancer [39].

We could not validate 11p15.5 loss with our real-time PCR method that was used to show the amplification of 8q21.13. The development of an efficient methodology that can validate the copy number loss should be given priority in future studies.

Ragnarsson et al. [40] found a high percentage of LOH at 1p36 and significant separation of survival curves between breast cancer patients with and without this alteration. These researchers also showed that LOH at 1p is a better prognostic indicator than any other variable, including lymph node metastasis.

Chromosome 8q gain is known to be associated with poor outcome in patients with clinically localized prostate cancer [41,42] and node-negative breast cancer [30,31]. *TPD52 *on chromosome 8q21 has been proposed as a potential amplification target gene in this region [43-45].

We divided the patients into axillary lymph node negative and positive breast cancers to avoid biases caused by mixing the different risk groups, because the axillary lymph node status has been shown to be the single most important prognostic factor for disease-free survival and overall survival in breast cancer patients. Due to the small number of cases in each group, no chromosomal aberration was different in frequency between the two risk groups. 1p36 and 11p15, the most significant chromosomal location in overall analysis, were also found more in Recurrence group than Non-recurrence group with modest significance in lymph node positive patients. More cases are required to confirm this result in the subgroup analysis and to elucidate the usefulness of these potential prognostic factors in lymph node negative patients.

In the recent publication of Arpino et al., ER+/PR- tumors express higher levels of HER-1 and HER-2 and display more aggressive features than ER+/PR+ tumors [46]. In this study, we showed that several chromosomal aberrations, such as 11p15.5 loss have stronger effect on distant recurrence of ER+ patients than PR status. It would be also interesting to stratify the patients according to PR expression status and investigate whether the pattern of DNA copy change is different between ER+/PR- and ER+/PR+ tumors.

A major weakness of our study is the small number of samples used, which was due to the limited availability of suitable fresh-frozen breast cancer tissues. DNA from formalin-fixed, paraffin-embedded tissues that had been archived for many years was tested, but it was so fragmented that it could not be utilized in this array CGH study.

With the increasing needs for efficient software that automatically select regions of gains and losses in array CGH studies, many algorithms have been recently developed [17-19,47]. We used a representative software and algorithm, CGH-Explorer and ACE, for thresholding the copy number ratios. It was comprehensive and had user-friendly graphical tools. An analytical tool that can identify the clones between two subject groups has not yet been established for array CGH.

## Conclusion

In conclusion, using array CGH analysis with BAC clones, we were able to detect various genomic alterations in ER-positive breast cancers. Patients in the Recurrence group showed a significantly different pattern of chromosomal gain and loss than patients in the Non-recurrence group. Copy number loss of 11p15 and 1p36 and gain of 8q21 are significantly associated with distant recurrence of the disease within 5 years of diagnosis. These regions will be further explored for the identification of metastasis-suppressing or -enhancing genes using a higher resolution method with a larger number of study subjects.

## Competing interests

The author(s) declare that they have no competing interests.

## Authors' contributions

WH selected cases, reviewed medical records, analyzed data, and drafted the manuscript. MRH and YJB did statistical analysis. JJK and JHL carried out array CGH and real time PCR experiment. JYB stored specimens and extracted genomic DNA. JEL, HJS, KTH, SEH, SWK, and DYN conceived of the study, and participated in its design and coordination. All authors read and approved the final manuscript.

## Pre-publication history

The pre-publication history for this paper can be accessed here:



## Figures and Tables

**Table 1:** Clinicopathological characteristics of 28 patients with estrogen receptor-positive invasive ductal carcinoma

Characteristic	Recurrence group N (%)	Non-recurrence group N (%)	*p *value
Age (range)	41.0 (24–62)	43.1 (29–65)	0.576
T stage			
T1	2 (22.2)	2 (10.5)	
T2	7 (77.8)	17 (89.5)	0.409
Lymph node metastasis			
Negative	2 (22.2)	6 (31.6)	
Positive	7 (77.8)	13 (68.4)	0.609
Histological grade			
1–2	4 (44.4)	11 (57.9)	
3	5 (55.6)	8 (42.1)	0.505
Nuclear grade			
1–2	4 (44.4)	7 (36.8)	
3	5 (55.6)	12 (63.2)	0.700
PR			
Positive	6 (66.7)	14 (73.7)	
Negative	3 (33.3)	5 (26.3)	0.701

**Table 2:** Most frequent genomic aberrations (>20%) in 28 estrogen receptor-positive invasive ductal carcinomas

Gain (core*)	Frequency (%)^†^	Loss (core)	Frequency (%)
1q21.1-q44 (q32.1)	50.0–71.4	6q11.1-q27 (q14.1-16.3)	28.6–42.9
17q23.2-q25.3 (q23.3)	57.1–67.9	11q13.5-q24.3 (q21-24.3)	21.4–42.9
8q11.21-q24.3 (q24.11)	42.9–64.3	13q12.11-q33.3 (q13.2-14.3)	25.0-39.3
17q12-q22 (q12-21.1)	35.7–53.6	1p32.3-p13.3 (p22.3-21.1)	21.4–32.1
8p12-p11.1 (p11.21)	35.7–42.9	4q12-q35.2 (q13.1-34.3)	21.4–32.1
19p13.3-p13.11 (p13.12-13.11)	35.7–39.3	22q11.21-q12.3 (q11.21)	25.0–32.1
19q12-q13.42 (q12-13.2)	35.7–39.3	3p21.1-p13 (p14.3-14.1)	21.4–28.6
20q11.21-q13.33 (q13.13-13.32)	25.0–39.3	4p15.32-4p12	28.6
5p15.33	35.7	6p12.3-p11.2 (p12.3)	25.0–28.6
16p13.3-p11.2	35.7	9p24.3-p21.2 (p22.3-21.2)	21.4–28.6
3q24-q29 (q29)	21.4–32.1	5q21.2-q21.3	25.0
22q11.21-q13.33 (q11.21)	21.4–32.1	8p23.2-p21.1 (8p22-21.1)	21.4-25.0
1p36.33-p35.3 (1p36.33)	21.4–28.6	11p15.5-p15.2 (p15.4-15.2)	21.4-25.0
9q33.3-q34.3 (q34.11-34.3)	25.0–28.6	11p11.2-p11.12	25.0
20p11.22-p11.21 (p11.21)	25.0–28.6	14q24.3-q31.3 (q31.1-31.3)	21.4–25.0
11q13.3	25.0–28.6	10q23.31-q25.3	21.4
1p35.3	25.0	17p13.3-p11.2	21.4
5p15.32-p12 (p13.2-13.1)	21.4–25.0	22q13.2-13.33	21.4
13q34	25.0		
16q24.3	25.0		
3q12.1-q13.33	21.4		
7q22.1	21.4		
7q36.3	21.4		
17p11.2	21.4		

*Region of the highest frequency of alteration within a given range of gain or loss regions

^†^Percentage of patients who have gain/loss. When an aberration covers more than one BAC clone, and the frequencies are uneven across the BAC clones, the minimum and maximum frequencies are shown.

**Table 3:** BAC clones and chromosomal location with significant differences in frequency of gain between patients in the Recurrence and Non-recurrence groups

BAC clone	Chromosome location	Representative genes	No. of gains in the Recurrence group	No. of gains in the Non-recurrence group	Adjusted *p *value
BAC217_O23	8q21.13	IL7	8	6	0.005
BAC178_P02	8q21.13		8	6	0.005
BAC232_P18	19p13.12	CHERP, SLC35E1, CRSP7	0	10	0.007
BAC246_J06	19p13.11	MAST3, PIK3R2, IFI30, RAB3A	0	10	0.007
BAC192_L06	19p13.11	FKBP8, UBA52, CRLF1	0	10	0.007
BAC195_K16	19p13.11	ZNF253, ZNF505	0	10	0.007
BAC144_L05	19q12		0	10	0.007
BAC90_H02	19q13.12	FXYD5, LISCH7, USF2, HAMP	0	10	0.007
BAC162_E14	19q13.13	HKR1, ZNF527, ZNF569	0	10	0.007
BAC162_K04	19q13.2	PD2, IXL, ZFP36, RPS16, SUPT5H	0	10	0.007
BAC146_F01	19q13.2	MAP3K10, AKT2	0	10	0.007
BAC162_J04	19q13.2	SNRPA, MIA, RAB4B, EGLN2, CYP2T2P, CYP2F1P	0	10	0.007
BAC249_F13	19q13.2	CYP2S1, AXL, HNRPUL1	0	10	0.007
BAC239_N12	19q13.31	XRCC1, ZNF576, IGSF4C, PLAUR	0	10	0.007
BAC183_D06	19q13.31	PLAUR, R30953_1, KCNN4	0	10	0.007
BAC78_D10	19q13.31	PLAUR, R30953_1	0	10	0.007
BAC233_C05	19q13.32	FOSB, RTN2, VASP, OPA3	0	10	0.007
BAC130_J22	19q13.41	KLK1, KLK15, KLK3, KLK2	0	10	0.007
BAC53_O15	19q13.42	NALP12	0	10	0.007
BAC168_E17	19q13.42		0	10	0.007
BAC8_P07	5q13.1		3	0	0.008
BAC12_H03	5q14.3	RASA1	3	0	0.008
BAC237_N24	5q15		3	0	0.008
BAC13_G12	5q15		3	0	0.008
BAC147_D19	5q15		3	0	0.008
BAC185_O16	5q11.2		3	0	0.008
BAC171_A01	5q11.2		3	0	0.008
BAC135_P04	5q11.2		3	0	0.008
BAC144_P08	5q12.1	DEPDC1B	3	0	0.008
BAC140_C04	5q12.3	SDCCAG10	3	0	0.008
BAC166_A05	5q13.2	RAD17, MARVELD2	3	0	0.008
BAC154_I09	5q13.2	FCHO2	3	0	0.008
BAC119_I19	5q13.3	ANKRD31	3	0	0.008
BAC96_B13	5q13.3	PDE8B, WDR41	3	0	0.008
BAC12_E06	5q14.2	RPS23	3	0	0.008
BAC25_M19	5q14.3		3	0	0.008
BAC152_G16	8q12.1		7	5	0.01
BAC139_M03	8q24.22	SIAT4A	8	7	0.01
BAC90_C04	8q24.22		8	7	0.01
BAC76_N02	8q24.23-24.3	COL22A1	8	7	0.01
BAC145_H17	8q11.21		7	5	0.01

**Table 4:** BAC clones and chromosomal location with significant differences in deletion frequency between patients in the Recurrence and Non-recurrence groups

BAC clone	Chromosome location	Representative genes	No. of losses in the Recurrence group	No. of losses in the Non-recurrence group	Adjusted *p *value
BAC165_D01	11p15.5	RIC8A, SIRT3, PSMD13, NALP6, MRPS24P1, IFITM2	6	1	<0.001
BAC121_J14	11p15.5	TOLLIP, STK29	6	1	<0.001
BAC60_J23	11p15.4	KCNQ1, CDKN1C, KCNQ1DN, SLC22A1LS, SLC22A18	6	1	<0.001
BAC252_K02	11p15.4	PHLDA2, NAP1L4	6	1	<0.001
BAC193_P15	11p15.4	NUP98, FRAG1, RHOG, STIM1	6	1	<0.001
BAC121_J11	11p15.4	SMPD1, APBB1, HPX	6	1	<0.001
BAC91_J13	11p15.4	TRIM3, ARFIP2, FXC1	6	1	<0.001
BAC88_B15	11p15.3	USP47	6	2	0.002
BAC82_C10	11p15.3-15.2		6	2	0.002
BAC35_I01	11p15.2	PDE3B, CYP2R1, CALCP	6	2	0.002
BAC219_F22	11p15.4	ILK, TAF10, CLN2, PCDH16	6	2	0.002
BAC96_J11	11p15.4	TUB, RIC3	6	2	0.002
BAC195_A16	11p15.4	STK33	6	2	0.002
BAC32_K13	11p15.4	ST5	6	2	0.002
BAC185_G10	11p15.4	C11orf16, ASCL3, C11orf15, NRIP3, SCUBE2	6	2	0.002
BAC252_G23	11p15.4	XLKD1, MRVI1	6	2	0.002
BAC96_G07	11q13.4	POLD3, CHRDL2	5	1	0.002
BAC15_I17	22q12.3-13.1	TMPRSS6, IL2RB, C1QTNF6, SSTR3, RAC2	3	0	0.008
BAC230_P11	1p36.33	SLC35E2	3	0	0.008
BAC101_I16	1p36.33		3	0	0.008
BAC176_F17	1p36.33	PRKCZ, SKI	3	0	0.008
BAC38_N15	1p36.33	SKI	3	0	0.008
BAC131_H09	22q12.3	MYH9	3	0	0.008
BAC141_N19	1p36.32	WDR8, TP73,	3	0	0.008
BAC120_F15	1p35.3	EYA3	3	0	0.008
BAC171_P09	1p36.32		3	0	0.008
BAC90_A08	1p36.22	FRAP1, CDT6, TERE1	3	0	0.008
BAC180_C01	1p36.22	AGTRAP, DKFZp434E1410, MTHFR, CLCN6	3	0	0.008
BAC91_A07	1p36.21		3	0	0.008
BAC80_C03	1p36.13	SHARP, ZBTB17, HSPB7, CLCNKA, CLCNKB	3	0	0.008
BAC68_M07	1p36.12	ALPL	3	0	0.008
BAC133_M03	1p36.12	RAP1GA1, USP48	3	0	0.008
BAC13_F18	1p36.12	CDC42, WNT4	3	0	0.008
BAC236_J05	1p36.11	MPRA, STMN1	3	0	0.008
BAC37_K05	1p36.11-35.3	FGR, G1P3, STX12	3	0	0.008
BAC46_E05	1p35.3	EYA3	3	0	0.008
BAC116_D07	11q13.5	UVRAG	5	2	0.010
BAC222_F09	11q13.5	C11orf30	5	2	0.010
BAC144_K18	11q13.5	GARP	5	2	0.010
BAC195_B24	17p13.1	ASGR1, DLG4, ACADVL, DVL2, GABARAP, DULLARD, DERP6, CLDN7, SLC2A4, YBX2, EIF5A, GPS2	5	2	0.010

**Table 5:** Most significant BAC clones that differentiate patients in the Recurrence group from those in the Non-recurrence group by SAM analysis

BAC Clone	Chromosome Location	Representative genes	*d*	Fold Change	*q *value (%)
BAC165_D01	11p15.5	RIC8A, SIRT3, PSMD13, NALP6, MRPS24P1, IFITM2	-4.537	0.817	0.339
BAC121_J14	11p15.5	TOLLIP, STK29	-4.015	0.795	0.339
BAC60_J23	11p15.4	KCNQ1, CDKN1C, KCNQ1DN, SLC22A1LS, SLC22A18	-3.943	0.774	0.339
BAC101_I16	1p36.33	SLC35E2	-3.872	0.799	0.339
BAC144_G04	7q22.1	PDAP1, G10, CPSF4, PTCD1, ATP5J2	-3.804	0.860	0.339
BAC193_P15	11p15.4	NUP98, FRAG1, RHOG, STIM1	-3.676	0.811	0.339
BAC252_K02	11p15.4	CDKN1C, KCNQ1DN, SLC22A1LS, SLC22A18, PHLDA2, NAP1L4	-3.566	0.800	0.339
BAC176_F17	1p36.33	PRKCZ, SKI	-3.561	0.799	0.339
BAC121_J11	11p15.4	SMPD1, APBB1, HPX	-3.531	0.844	0.339
BAC228_L09	11q13.1	ESRRA, HSPC152, PRDX5, RPS6KA4	-3.513	0.828	0.339
BAC33_L13	19p13.3	NFIC, FZR1, C19orf28	-3.486	0.804	0.339
BAC75_B14	19p13.3	EDG6, BRUNOL5	-3.335	0.817	0.339
BAC146_F01	19q13.2	MAP3K10, AKT2	-3.327	0.847	0.339
BAC249_F13	19q13.2	CYP2S1, AXL, HNRPUL1	-3.288	0.869	0.339
BAC176_M06	9q34.3	TRAF2, FBXW5, C8G, LCN12	-3.286	0.862	0.339
BAC82_M18	3q21.2	SLC12A8	-3.271	0.913	0.339
BAC162_J04	19q13.2	SNRPA, MIA, RAB4B, EGLN2, CYP2T2P, CYP2F1P	-3.266	0.862	0.339
BAC91_J13	11p15.4	TRIM3, ARFIP2, FXC1	-3.224	0.853	0.339
BAC96_L08	19p13.3	PPAP2C, THEG	-3.195	0.860	0.339
BAC185_L04	11q13.1	SF1, MAP4K2, MEN1, HSMDPKIN, EHD1, KIAA0404, PPP2R5B	-3.182	0.833	0.339
BAC168_E17	19q13.42	ZNF579, ZNF524	-3.182	0.872	0.339
BAC65_N13	19p13.3	TJP3, APBA3, MRPL54, MATK	-3.175	0.825	0.339
BAC224_C15	9q34.3	MAN1B1, DPP7, GRIN1	-3.128	0.857	0.339
BAC42_H14	10q26.2-26.3		-3.127	0.863	0.339
BAC122_I18	11q13.1	NAALADL1, CDCA5, ZFPL1, C11orf2, TM7SF2, ZNHIT2, FAU, MRPL49, HRD1	-3.126	0.846	0.339
BAC37_C21	19p13.3	ANKRD24, EBI3, SHD, FSD1, STAP2, SH3GL1	-3.116	0.840	0.339
BAC167_H05	11p11.2	KAI1	-3.112	0.883	0.339
BAC29_C11	2p25.1	HPCAL1, ODC1	-3.084	0.867	0.339
BAC195_K21	11q12.3	EEF1G, RBM21	-3.060	0.868	0.339
BAC80_E04	21q22.3	UBE2G2, SUMO3, PTTG1IP	-3.053	0.854	0.339
BAC192_C22	11q13.1	TIGD3, FKSG44	-3.051	0.875	0.339
BAC57_D10	19p13.3	PRSS15, FUT6, NRTN	-3.044	0.858	0.339
BAC76_D18	2p25.1	HPCAL1, ODC1	-3.024	0.907	0.339
BAC30_K23	19q13.32		-3.022	0.890	0.339
BAC128_J04	11q13.1	BANF1, CST6, CATSPER1, GAL3ST3, SF3B2, PACS1	-2.997	0.879	0.339
BAC76_C20	5q35.3	RASGEF1C	-2.994	0.884	0.339
BAC215_J18	1p34.2	MYCL1	-2.976	0.896	0.339
BAC63_I09	1p35.3		-2.959	0.886	0.339
BAC78_D10	19q13.31	PLAUR	-2.950	0.875	0.339
BAC132_L20	17p11.2	FBXW10, FAM18B	-2.948	0.877	0.339
BAC104_F07	6p21.32	CREBL1, FKBPL, C6orf31, PPT2, EGFL8, AGPAT1, RNF5, AGER, PBX2, GPSM3, NOTCH4	-2.947	0.850	0.339
BAC38_N15	1p36.33	SKI	-2.939	0.875	0.339
BAC209_K24	21q22.3	PTTG1IP, ITGB2, C21orf69, C21orf67, C21orf70	-2.931	0.853	0.339
BAC136_D13	21q22.3	UBE2G2, SUMO3, PTTG1IP	-2.894	0.869	0.339
BAC252_I22	16p13.3	TSC2, PKD1, TRAF7, RAB26	-2.881	0.820	0.339
BAC30_F19	7q32.2	UBE2H	-2.873	0.901	0.339
BAC197_D09	11q12.2	PGA5	-2.872	0.874	0.339
BAC183_D06	19q13.31	RIC-8, SIRT3, PSMD13, NALP6, MRPS24P1, IFITM2	-2.839	0.891	0.339
BAC239_N12	19q13.31	TOLLIP, STK29	-2.835	0.889	0.339
BAC120_F15	1p35.3	KCNQ1, CDKN1C, SLC22A1LS, SLC22A18	-2.833	0.908	0.339
BAC219_C01	6p21.33	SLC35E2	-2.824	0.857	0.339
BAC91_A07	1p36.21	PDAP1, G10, CPSF4, PTCD1, ATP5J2	-2.822	0.888	0.339
BAC59_B08	16p13.3	NUP98, FRAG1, RHOG, STIM1	-2.809	0.854	0.339
BAC1_C02	21q22.3	CDKN1C, KCNQ1DN, SLC22A1LS, SLC22A18, PHLDA2, NAP1L4	-2.804	0.878	0.339
BAC142_E13	9q34.2	PRKCZ, SKI	-2.803	0.888	0.339
BAC100_L06	7q11.22	SMPD1, APBB1, HPX	-2.803	0.927	0.339
BAC149_E07	11q13.2	ESRRA, HSPC152, PRDX5, RPS6KA4	-2.801	0.886	0.339
BAC10_E08	15q25.1	NFIC, FZR1, C19orf28	-2.797	0.895	0.339
BAC232_G07	13q34	EDG6, BRUNOL5	-2.780	0.895	0.339
BAC155_O07	21q22.3	MAP3K10, AKT2	-2.773	0.875	0.339
BAC219_N21	2q14.3	CYP2S1, AXL, HNRPUL1	-2.762	0.912	0.339
BAC24_E16	10q22.2	TRAF2, FBXW5, C8G, LCN12	-2.751	0.867	0.339
BAC142_B18	9q34.11	SLC12A8	-2.747	0.873	0.339
BAC168_H24	19q13.33	SNRPA, MIA, RAB4B, EGLN2, CYP2T2P, CYP2F1P	-2.745	0.912	0.339
BAC233_C05	19q13.32	TRIM3, ARFIP2, FXC1	-2.744	0.905	0.339
BAC70_M18	10p15.3	PPAP2C, THEG	-2.718	0.880	0.339
BAC32_K13	11p15.4	SF1, MAP4K2, MEN1, HSMDPKIN, EHD1, KIAA0404, PPP2R5B	-2.712	0.907	0.339
BAC127_P09	12q13.13	ZNF579, ZNF524	-2.696	0.929	0.339
BAC104_G21	5p15.33	TJP3, APBA3, MRPL54, MATK	-2.690	0.917	0.339
BAC110_G12	19p13.3	MAN1B1, DPP7, GRIN1	-2.676	0.879	0.339
BAC125_M06	9q34.13		-2.668	0.880	0.339
BAC4_J24	9q34.3	NAALADL1, CDCA5, ZFPL1, C11orf2, TM7SF2, ZNHIT2, FAU, MRPL49, HRD1	-2.663	0.890	0.339
BAC142_G12	16p13.3	ANKRD24, EBI3, SHD, FSD1, STAP2, SH3GL1	-2.657	0.826	0.339
BAC100_M24	6p21.32	KAI1	-2.656	0.896	0.339
BAC195_F16	7q32.1	HPCAL1, ODC1	-2.655	0.913	0.339
BAC109_M06	7q22.1	EEF1G, RBM21	-2.653	0.916	0.339
BAC247_C06	3q21.3	UBE2G2, SUMO3, PTTG1IP	-2.645	0.935	0.339

BAC140_C04	5q12.3	TIGD3, FKSG44	3.601	1.104	0.686
BAC86_O11	20p12.1	PRSS15, FUT6, NRTN	3.435	1.160	0.686
BAC125_H18	2p11.2	HPCAL1, ODC1	3.308	1.119	0.686
BAC122_F23	7p14.1		3.295	1.131	0.686
BAC89_K07	7p14.2	BANF1, CST6, CATSPER1, GAL3ST3, SF3B2, PACS1	3.286	1.146	0.686
BAC69_J21	2p12	RASGEF1C	3.244	1.097	0.686
BAC24_A20	7p14.2	MYCL1	3.236	1.135	0.686
BAC165_M10	20p12.1		3.601	1.104	0.686
BAC140_C04	5q12.3	PLAUR	3.435	1.160	0.686
BAC86_O11	20p12.1	FBXW10, FAM18B	3.308	1.119	0.686
BAC125_H18	2p11.2	CREBL1, FKBPL, C6orf31, PPT2, EGFL8, AGPAT1, RNF5, AGER, PBX2, GPSM3, NOTCH4	3.295	1.131	0.686
BAC122_F23	7p14.1	SKI	3.286	1.146	0.686
BAC89_K07	7p14.2	PTTG1IP, ITGB2, C21orf69, C21orf67, C21orf70	3.244	1.097	0.686
BAC69_J21	2p12	UBE2G2, SUMO3, PTTG1IP	3.236	1.135	0.686
BAC24_A20	7p14.2	TSC2, PKD1, TRAF7, RAB26	3.601	1.104	0.686

**Table 6:** Multivariate analysis for predictors of distant recurrence based on the Cox proportional hazards regression model

Variables	Hazard ratio	95% Confidence interval	*p *value
11p15.5 loss	12.3	2.74–55.44	.001
Tumor size ≥ 2 cm	1.0	0.17–6.12	.965
Lymph node-positive	2.0	0.30–14.28	.457
Nuclear grade 3	3.3	0.40–28.19	.264
PR	1.5	0.38–6.46	.533
Age<35 years	0.9	0.87–1.04	.327

**Table 7:** Chromosomal locations showing different frequencies of aberration between the two groups in lymph node positive patients

	Recurrence group (N = 7)		Non-recurrence group (n = 13)		Adjusted *p *value
			
	Aberration	No aberration	Aberration	No aberration	
1p36.33-1p35.3 loss	3	4	0	13	0.052
4q13.1-4q34.3 loss	0	7	7	6	0.052
11p15.5-11p15.4 loss	4	3	1	12	0.052
19q12-19q13.2 gain	0	7	8	5	0.052
8q21.13-8q24.22 gain	6	1	5	8	0.111
11p11.2-11p11.12 loss	4	3	2	11	0.112
17p13.3-17p11.2 loss	3	4	1	12	0.113
7p21.2-7p13 gain	3	4	2	11	0.286
11q14.3-11q24.3 loss	4	3	4	9	0.326
17q23.2-17q24.2 gain	3	4	9	4	0.326
20q13.13-20q13.33 gain	3	4	3	10	0.421
1q32.1-1q44 gain	6	1	9	4	0.451
